# Expression of striated activator of rho‐signaling in human skeletal muscle following acute exercise and long‐term training

**DOI:** 10.14814/phy2.13624

**Published:** 2018-03-04

**Authors:** Stefan M. Reitzner, Jessica Norrbom, Carl Johan Sundberg, Eva‐Karin Gidlund

**Affiliations:** ^1^ Department of Physiology and Pharmacology Karolinska Institutet Stockholm Sweden; ^2^ Department of Learning, Informatics, Management and Ethics Karolinska Institutet Stockholm Sweden

**Keywords:** Acute exercise, endurance, gene expression, human, long‐term training, PGC‐1*α*, protein expression, STARS

## Abstract

The striated activator of rho‐signaling (STARS) protein acts as a link between external stimuli and exercise adaptation such as muscle hypertrophy. However, the acute and long‐term adaptational response of STARS is still unclear. This study aimed at investigating the acute and long‐term endurance training response on the mRNA and protein expression of STARS and its related upstream and downstream factors in human skeletal muscle. mRNA and protein levels of STARS and related factors were assessed in skeletal muscle of healthy young men and women following an acute bout of endurance exercise (*n* = 15) or 12 weeks of one‐legged training (*n* = 23). Muscle biopsies were obtained before (acute and long‐term), at 30 min, 2, and 6 h following acute exercise, and at 24 h following both acute exercise and long‐term training. Following acute exercise, STARS mRNA was significantly elevated 3.9‐fold at 30 min returning back to baseline 24 h after exercise. STARS protein levels were numerically but nonsignificantly increased 7.2‐fold at 24 h. No changes in STARS or ERR *α* mRNA or STARS protein expression were seen following long‐term training. PGC‐1*α* mRNA increased 1.7‐fold following long‐term training. MRTF‐A mRNA was increased both following acute exercise and long‐term training, in contrast to SRF mRNA and protein which did not change. STARS mRNA is acutely upregulated with exercise, but there is no cumulative effect to long‐term training as seen in PGC‐1*α* mRNA expression. Exercise intensity might play a role in manifestation of protein expression, suggesting a more complex regulation of STARS.

## Introduction

Physical activity is known to cause adaptations in tissues and systems such as skeletal muscle (Holloszy and Booth 1967), tendons and bone (Russo 2009; Daly 2010), cardiovascular system (Golbidi and Laher 2012), and the central nervous system (Morgan et al. 2015). Additionally, physical activity can induce beneficial effects against diseases like type 2 diabetes (Kelley et al. 2002; Earnest 2008), cardiovascular disease and cancer (Rundqvist et al. 2013; Mijwel et al. 2017). Skeletal muscle is a key tissue in physical activity, being strongly activated, directly influencing, for example, glucose control, blood pressure regulation and possibly indirectly, through release of myokines, adipose tissue function. Generally, the structural and functional adaptations of regular physical activity that lead to health and performance improvements are mediated through biophysical, biochemical, and physiological stimuli. The molecular mechanisms mediating the adaptational processed are poorly understood. The protein striated activator of rho signaling (STARS), also referred to as ABRA (Troidl et al. 2009) (Actin‐binding rho activating protein) or MS1 (Mahadeva et al. 2002) (Myocyte stress‐1) has emerged as a tentative link between the mechanical stimuli and the cellular response‐ and adaptation‐processes in skeletal muscle (Arai et al. 2002).

STARS is found in striated muscles tissue like skeletal and cardiac muscle, localized to the actin‐binding regions in the sarcomere (Arai et al. 2002). Together with RhoA, STARS has been suggested to promote organization of monomeric G‐Actin into structured, polymeric F‐Actin (Arai et al. 2002). Through this process, G‐Actin‐induced inhibition of myocardin‐related transcription factor‐A (MRTF‐A) is removed, enabling nuclear translocation (Sotiropoulos et al. 1999), which in turn has been shown to increase the transcriptional coactivation of the serum response factor (SRF) (Miralles et al. 2003). An increase in SRF permits activation of downstream targets known for their role in skeletal muscle mass maintenance, growth, and metabolism (Charvet et al. 2006). Examples of such downstream targets include the myogenic regulation factor MyoD (Ounzain et al. 2008), the myocyte enhancer factor‐2 (MEF2) (Kuwahara et al. 2007), but also β‐oxidation enzymes like carnitine palmitoyltransferase‐1*β* (CPT‐1*β*) and transcription factors responsible for growth regulation like JunB (Wallace et al. 2011). SRF, MEF2, and MyoD also act as transcriptional regulators of STARS, thereby eliciting a feedback‐loop mechanism (Wallace et al. 2012). Furthermore, STARS has also been identified as a target of several other well studied factors with particular importance in exercise adaptation, such as the estrogen related receptor alpha (ERR*α*), a nuclear receptor important in mitochondrial biogenesis (Wu et al. 1999) and energy metabolism (Yoon et al. 2001; Huss et al. 2004; Mootha et al. 2004), and the peroxisome proliferator‐activated receptor gamma coactivator 1 alpha (PGC‐1*α*) (Wallace et al. 2011), a well‐known regulator of exercise‐induced mitochondrial biogenesis (Pilegaard et al. 2003; Pilegaard and Richter 2008; Sanchis‐Gomar et al. 2014). Together, ERR*α* and PGC‐1*α* form a transcriptional complex with a binding site located 150 basepairs upstream of the transcriptional start site of STARS (Wallace et al. 2011). Both have been shown to be influenced by physical activity and regulate genes involved in skeletal muscle function (Cartoni et al. 2005; Russell et al. 2005; Hock and Kralli 2009; Wallace et al. 2011). Furthermore, in C2C12 myotubes, the STARS gene has also been shown to be a target of this transcriptional PGC‐1*α*/ERR*α* complex (Wallace et al. 2011), again supporting a potential regulatory mechanism and connection to STARS.

The exercise‐induced response of both PGC‐1*α* and ERR*α* vary dependent on the exercise type performed as well as exercise intensity, duration and/or frequency (Ydfors et al. 2013). Previous studies have suggested that basal PGC‐1*α* expression in human skeletal muscle varies between the different muscle fiber types (Russell et al. 2003), and that specific fiber‐type composition profiles might be beneficial to certain sports (Klitgaard et al. 1990; D'Antona et al. 2006; Trappe et al. 2006; Farup et al. 2014). Additionally, PGC‐1*α* seems to be highly responsive to hypoxic stress (Arany et al. 2008), *β*‐adrenergic signaling or supplementation of *β*‐adrenergic receptor agonists (Chinsomboon et al. 2009; Brandt et al. 2017). A recent study found differences in PGC‐1*α* protein expression following either continuous exercise training, high intensity interval training (HIIT) or sprint interval training, and somewhat surprisingly only sprint interval training induced an increased PGC‐1*α* protein expression (Granata et al. 2016). Furthermore, ERR*α* and PGC‐1*α* have the potential to elicit specific acute and long‐term training patterns of expression (Baar et al. 2002; Lin et al. 2002; Norrbom et al. 2004; Wallace et al. 2011; Egan et al. 2013). A study by Perry et al. (2010) demonstrated that, in a 2‐week training program (7 sessions in total), the acute increase of PGC‐1*α* mRNA expression following each bout of exercise significantly decreased over time. Thus, since STARS is regulated by both PGC‐1*α* and ERR*α*, it might be speculated that STARS also has an exercise‐specific expression pattern similar to PGC‐1*α* and ERR*α*. In fact, previous studies have shown that STARS can be regulated both acutely (Wallace et al. 2011) and following long‐term resistance or endurance training (Lamon et al. 2009, 2013). However, research comparing the effects of acute and long‐term endurance training on a more complete STARS network, including ERR*α* and PGC‐1*α*, has not been conducted so far.

In light of previous findings, the regulation of STARS and of downstream targets such as MRTF‐A and SRF (Sotiropoulos et al. 1999), as mediators of the cellular response in the context of exercise adaptation, is of great interest. The integration of STARS into skeletal muscle signaling and adaptation events might suggest further involvement of factors such as MEF2, MyoD, and CPT‐1*β*. In summary, STARS appears to be a very interesting player for adaptation, and its expression might be strongly influenced by parameters such as the type, intensity and duration of the physical activity performed. The specific effect of these parameters might lead to a specific pattern of activation of the STARS network and its related factors, which together steer the adaptational processes leading to the muscle specific physical adaptations.

Accordingly, the main aim of this study was to investigate the effects of acute endurance exercise over a 24 h timecourse and long‐term endurance training on STARS mRNA and protein expression as well as factors upstream and downstream of STARS in human skeletal muscle. Furthermore, we also aimed to investigate a possible influence of sex on gene and protein expression of STARS and related factors.

## Material and Methods

Before the respective interventions, subjects were introduced to the experimental procedures, informed about the study outlines and their written consent was obtained. Both intervention studies were approved by the Stockholm regional ethics board under observance of the declaration of Helsinki.

### Study subjects and exercise protocol

**Figure 1:**
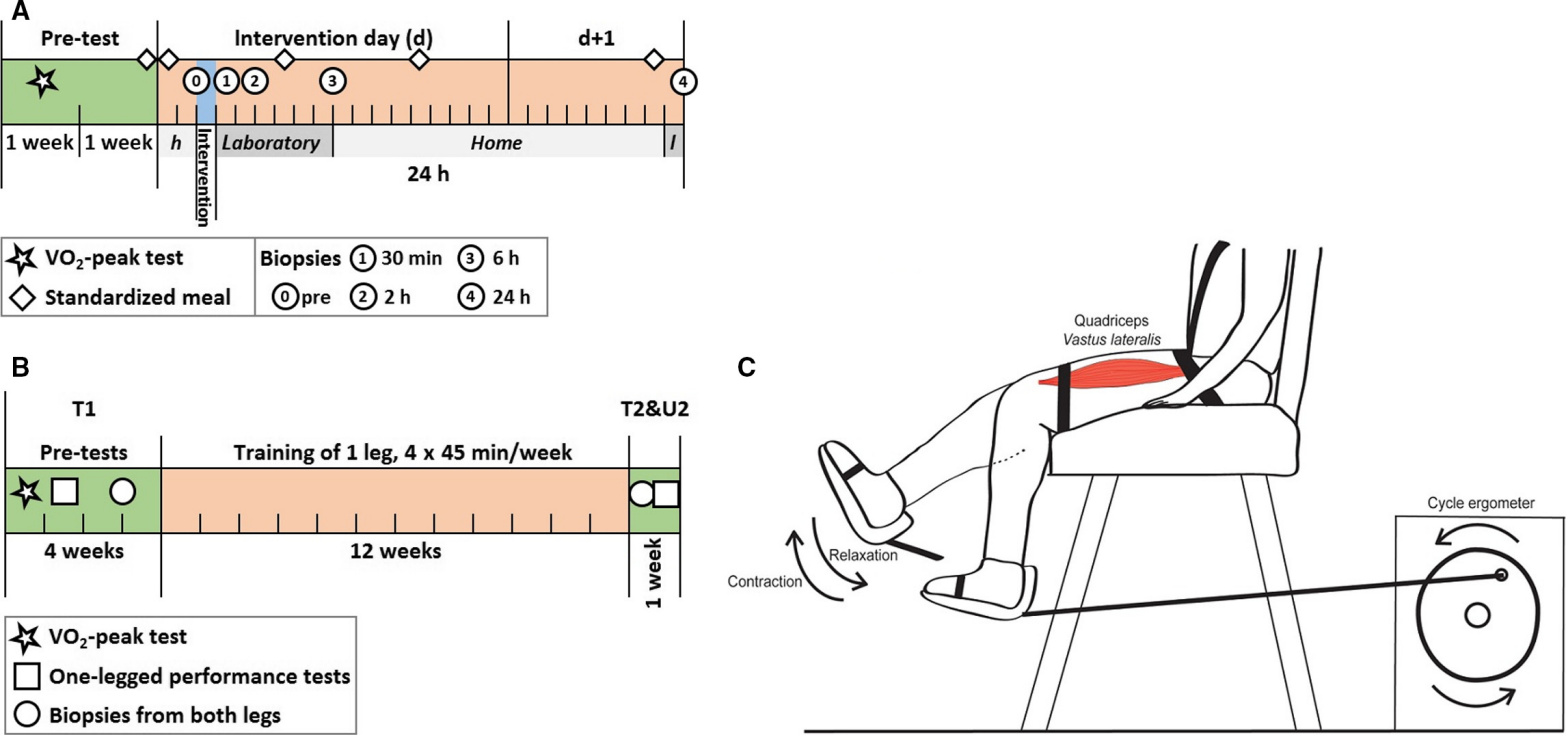
Study design of acute (A) and long‐term (B) intervention. Acute exercise consisted of 60 min of bipedal cycling on a cycle ergometer. Muscle biopsies from *M. vastus lateralis* were taken at five time points, before intervention (both legs) and 30 min, 2, 6, and 24 h after intervention (one leg). Food intake was standardized. Long‐term training was performed one‐legged on a leg extension machine connected to a cycle ergometer (C) for 12 weeks, subjects completed 45 exercise sessions in total. Muscle biopsies from *M. vastus lateralis* were taken before (T1) and after the training period from exercising and nonexercising leg (T2, U2). Subjects were asked to consume the same meal the evening and morning before each biopsy.

In the acute endurance exercise study (AE), 20 healthy, young nonsmoking subjects were recruited, 10 men and 10 women (Table 1). Subjects' peak oxygen uptake ($\dot{V}$O_2_peak) was measured using an incremental cycle ergometer test until exhaustion (for detailed protocol see (Gidlund et al. 2015)). In the AE study, subjects with $\dot{V}$O_2_peak <60 mL·kg^−1^·min^−1^ (men) and <50 mL·kg^−1^·min^−1^ (women) were included. Subjects were randomly assigned to exercise (7 men, 8 women) or control group (3 men, 2 women). There was no significant difference in age, weight, height, and $\dot{V}$O_2_peak between the groups. The exercise group performed 60 min of cycling exercise corresponding to 50% of their $\dot{V}$O_2_peak the first 20 min and 65% of their $\dot{V}$O_2_peak for the remaining 40 min, whereas the subjects of the control group were resting. To control for circadian rhythm effects, all subjects reported to the laboratory in the morning of the intervention and at the same time for the 24 h post testing. In the AE study all subjects were given standardized meals the night before, during the day of, and on the morning after the intervention (Fig. 1A).

**Table 1:** Baseline subject characteristics of the studies

	Age (years)	Height (cm)	Bodyweight (kg)	BMI (kg/m^2^)	$\dot{V}$O_2_peak (mL·kg^−1^·min^−1^)
Acute study					
Males (*n* = 10)	25.1 ± 2.6	181.7 ± 4.9	79.9 ± 6.6	24.2 ± 2.1	50.3 ± 4.6
Females (*n* = 10)	24.4 ± 2.8	168.5 ± 6.7	64 ± 6.5	22.7 ± 2.7	41.5 ± 3.6
All subjects (*n* = 20)	24.8 ± 2.7	175.1 ± 8.8	72.1 ± 10.3	23.5 ± 2.5	45.9 ± 6
Long‐term study					
Males (*n* = 12)	27.5 ± 3.4	180.8 ± 7.5	81.4 ± 18.2	24.8 ± 4.5	39.4 ± 3.7*
Females (*n* = 11)	26.4 ± 4.3	169.5 ± 6.2	66.87 ± 12	23.2 ± 3.1	38.3 ± 4.1*
All subjects (*n* = 23)	26.9 ± 3.8*	175.3 ± 8.9	74.5 ± 16.9	24.0 ± 3.9	38.9 ± 3.9*

Data are presented as mean ± SD. (*P < 0.05 for long‐term versus acute study).

For the long‐term endurance training study (LT), 23 young and healthy nonsmokers were recruited, 12 men and 11 women (Table 1), and underwent baseline testing of $\dot{V}$O_2_peak in the same way as in the acute exercise study and one‐legged knee extension‐tests, called 15‐min‐time‐trial (for detailed protocol see (Lindholm et al. 2014b)). After baseline measurements and biopsy, subjects progressively trained a randomized leg by one‐legged knee extension exercise (see Fig. 1C) four times per week for 12 weeks. Work load was increased based on testing after week 1, 2, 3, 5, and 6 (Lindholm et al. 2014b). All subjects completed 45 training sessions in total (Fig. 1B). Food diaries were kept on the day before pretesting and subjects were asked to repeat the same dietary intake on the day before posttesting. Furthermore, all subjects completed a one‐legged 15‐min‐time‐trial performance test after the training period (for detailed protocol see (Lindholm et al. 2014b)). The untrained leg was used as an intraindividual control.

### Muscle biopsies

In AE, skeletal muscle biopsies were taken from the left and right leg's *M. vastus lateralis* before exercise and at 30 min, 2, 6, and 24 h after a single exercise bout using the Bergström needle technique (Bergstrom 1975). Biopsies were obtained alternating from the left and right leg. Incisions were made proximal to the previous biopsy, separated by at least 2 cm to minimize the potential effect of local inflammation.

Subjects rested between biopsies, stayed in the laboratory until the 6 h post exercise biopsy and returned to the laboratory the next morning for the 24 h biopsy. The biopsy samples were snap frozen in liquid nitrogen and stored at −80°C.

In LT, skeletal muscle biopsies were taken from *M. vastus lateralis* of both, the leg randomized to training and the untrained leg, before the training period and 24 h after the last training session using the same Bergström needle technique as in the AE (Bergstrom 1975). All samples were snap frozen in liquid nitrogen and stored at ‐80°C.

### RNA extraction and cDNA synthesis

RNA from skeletal muscle was extracted using the phenol based TRIzol method (Invitrogen) and quantified spectrophotometrically on a NanoDrop 2000 (Thermo Scientific). RNA was reverse transcribed using Superscript Reverse Transcriptase (Fisher Scientific, 4368814).

### Real‐time PCR

RT PCR was performed in duplicates for the gene expression analysis of STARS, MRTF‐A, SRF, ERR*α*, PGC‐1*α,* and GAPDH (housekeeping) with a C1000 Touch thermal cycler (Bio‐Rad) using the TaqMan Gene expression Assay System (Applied Biosystems). TaqMan primers used were; STARS (ABRA, ms‐1; Hs00373623), MRTF‐A (MKL‐1; Hs00252979), SRF (Hs00182371), ERR*α* (ESSRA; HS01067166_g1), PGC‐1*α* (Hs01016724), and GAPDH (4352934E). All reactions were performed in TaqMan Fast Universal PCR Master Mix (Applied Biosystems, 4352042) in a cDNA dilution of 1:100 and a reaction size of 10 µl and loaded on 384‐well hard shell PCR plates (Bio‐Rad). Control and intervention group/leg measurements were performed together in random order.

### Protein extraction and western blot

Total protein was extracted using a glass homogenizer and RIPA‐buffer with a protease inhibitor cocktail (Roche Diagnostics) and centrifuged at 15 000 g for 10 min (for more details see (Gidlund et al. 2015)). 18 *µ*g protein was loaded on Tris‐Glycine PROTEAN Gels (Bio‐Rad) and blotted on nitrocellulose membrane using the Trans‐Blot Turbo system (Bio‐Rad). Primary Antibodies used were STARS (ABRA, ms‐1; ab116046, abcam), SRF (sc‐25290, Santa Cruz), PGC‐1*α* (38‐51; Capra Science, Ängelholm, Sweden), *α*‐Actinin (A7811, Sigma‐Aldrich), and *β*‐Tubulin (T5201, Sigma‐Aldrich). For secondary detection and quantification, the Odyssey system (Li‐Cor) and software was used together with corresponding IRDye‐based Li‐Cor antibodies anti‐mouse IgG (925‐32212), anti‐rabbit IgG (952‐32211), and anti‐chicken IgG (92532218). Control and intervention group/leg measurements were performed together in random order.

### Statistical analysis

In the acute study, statistics were calculated by two‐way ANOVA with post hoc testing using Dunnett's multiple comparison test (mRNA: *n* = 20; protein: *n* = 19(SRF)/11(STARS)). For the long‐term study, ANOVA was used together with Fisher's LSD post hoc test (mRNA: *n* = 15; protein: *n* = 23). Statistical differences in subject characteristics (age, height, weight, BMI, $\dot{V}$O_2_peak) were calculated using Student's *t*‐test. Correlation analyses of individual Δ and absolute mRNA expression were calculated using two‐tailed Pearson correlation coefficients. Statistics were calculated for male and female subjects separately, but pooled after no effect of sex could be detected. Differences were considered significant at *P* < 0.05. Results are presented as mean ± SEM unless stated otherwise. All results are displayed in fold changed, individually normalized to prevalue. Statistics and significance indicators on graphs (indicated by *) were calculated using nonnormalized data. Statistical outliers were identified and excluded combining an interquartile range test on prevalues with the intraindividual coefficient of variation. Based on this, two subjects were excluded from the analysis of PGC‐1*α* protein expression in LT. Statistical calculations were performed using IBM SPSS v23 and GraphPad Prism 7.03.

## Results

### STARS

**Figure 2:**
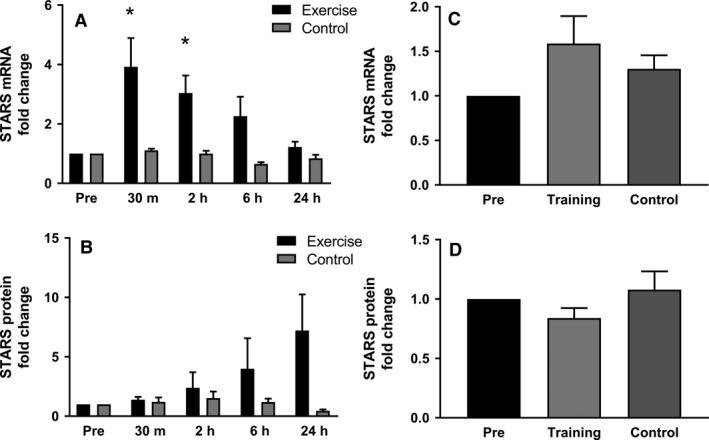
Influence of acute exercise (A and B) and long‐term training (C and D) on STARS mRNA and protein expression. (A) STARS mRNA is increased significantly as a response to acute exercise at the time points 30 min and 2 h compared to the pre time point and returns to baseline after 6 h. (B) STARS protein is increased 24 h after acute exercise. (C) Long‐term training does not influence STARS mRNA expression 24 h after the last training session. (D) STARS protein expression is not significantly influenced following long‐term training, 24 h after the last training session. Data are presented as mean ± SEM. (*P < 0.05).

STARS mRNA was significantly upregulated 3.9‐fold at 30 min and threefold at 2 h after acute endurance exercise (AE). Six hours after AE, a nonsignificant 2.6‐fold elevation in mRNA expression was found, whereas baseline expression was restored 24 h post exercise (Fig. 2A). Following a 12‐week endurance training program (LT), there was no difference in STARS mRNA expression 24 h after the last exercise session compared to baseline (Fig. 2C). Following AE, STARS protein expression showed a nonsignificant 7.2‐fold upregulation at the 24‐h time point (Fig. 2B). STARS protein expression did not change with LT (Fig. 2D). For individual results, see Figure S1.

### PGC‐1*α*

**Figure 3:**
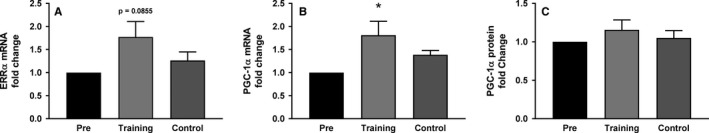
Upstream regulators of STARS, ERRα, and PGC‐1α regulation in response to long‐term training, 24 h after the last exercise. (A) ERRα is not significantly upregulated in response to training. A slight difference is visible comparing training and untrained leg after a training period. (B) PGC‐1α is significantly upregulated in the trained leg. A similar but nonsignificant tendency is also visible in the untrained leg. (C) Long‐term training does not elevate PGC‐1α protein expression 24 h after last exercise. Data are presented as mean ± SEM. (*P < 0.05).

PGC‐1*α* mRNA was significantly increased 1.8‐fold in the trained leg in response to LT. No change was found in the untrained leg (Fig. 3B). While, at the protein level, there were no significant changes (Fig. 3C). For individual results, see Figure S2. The response of PGC‐1*α* mRNA and protein to acute exercise has previously been published from this study material (Gidlund et al. 2015).

### ERR*α*

In the LT study, no significant change in ERR*α* mRNA expression could be detected (Fig. 3A). Several attempts to measure ERR*α* protein were performed, but no reliable results of ERR*α* protein could be quantified. For individual results, see Figure S2. The response of ERR*α* mRNA to acute exercise has been previously published from this study material (Gidlund et al. 2015).

### MRTF‐A

**Figure 4:**
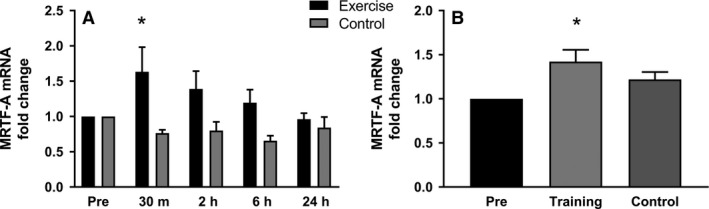
Regulation of MRTF‐A mRNA expression. (A) MRTF‐A mRNA expression is upregulated 30 min after end of one acute exercise bout. After that it gradually returns to base line. (B) In long‐term training, 24 h after last exercise, MRTF‐A is significantly upregulated in the trained leg but not in the untrained leg. (*P < 0.05).

MRTF‐A mRNA expression in AE followed a similar pattern to that of STARS. In response to AE, MRTF‐A expression showed a significant 1.6‐fold increase at 30 min, thereafter the levels were not significantly different from baseline (Fig. 4A). After LT, MRTF‐A mRNA was significantly upregulated by 1.4‐fold in the trained leg, and not affected in the untrained leg (Fig. 4B). For individual results, see Figure S3. Several attempts to measure MRTF‐A protein were performed but no reliable results could be quantified.

### SRF

**Figure 5:**
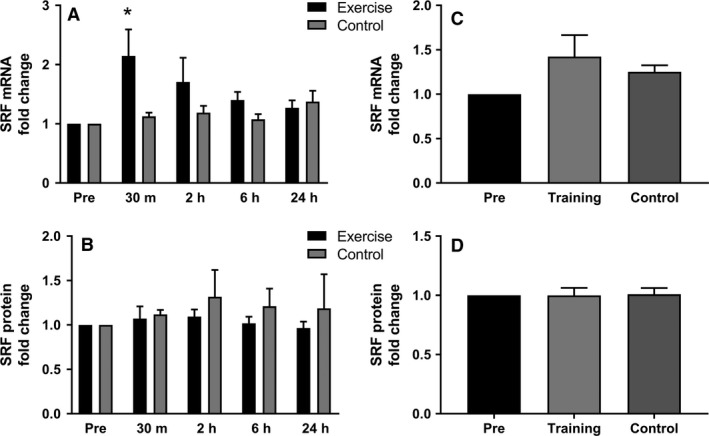
Influence of acute exercise (A and B) and long‐term training (C and D) on SRF mRNA and protein expression. (A) SRF mRNA is increased significantly as a response to acute exercise at time point 30 min compared to pre time point and returns to baseline after that. (B) SRF acute exercise protein expression is unchanged. (C) No statistically significant changes are visible in the trained and the untrained leg. (D) SRF protein expression remains unchanged 24 h following the last exercise in long‐term training. Data are presented as mean ± SEM. (*P < 0.05).

Expression of SRF mRNA was significantly increased 2.1‐fold following AE at 30 min, and returned to baseline 2 h post exercise (Fig. 5A). In LT, no significant changes in mRNA expression were found in the trained or the untrained leg (Fig. 5C). SRF protein expression was not affected following AE (Fig. 5B). For individual results see Figure S4. No significant changes in SRF protein could be detected in LT (Fig. 5D).

### Regression analyses

Regression analysis was performed for the difference between postexercise time points and baseline between ΔSTARS, ΔMRTF‐A, and ΔSRF mRNA in LT (Fig. S5A–C) and at the time points 30 min (Fig. S5D–F) and 2 h (Fig. S5G–H) in AE as well as for ΔPGC‐1*α* and ΔSTARS mRNA and protein expression between trained and untrained leg (Fig. S6).

In LT and at 30 min and 2 h in AE, ΔSTARS correlated with ΔMRTF‐A (Fig. S5A, D, G) and with ΔSRF only in AE (Fig. S5B, E, H). ΔSRF and ΔMRTF‐A correlated in LT and at 30 min and 2 h in AE (Fig. 5C, F, I). In LT, the change of expression over the training period in the trained and untrained legs, ΔSTARS and ΔPGC‐1*α* protein levels significantly correlated. Also, ΔSTARS mRNA levels in trained and untrained legs correlated significantly.

Furthermore, regression analysis of the performance markers $\dot{V}$O_2_peak and the one‐legged 15‐min‐time‐trial results in LT showed no correlation with either absolute PGC‐1*α* mRNA or protein levels at baseline or ΔPGC‐1*α* mRNA or protein expression in the trained leg (Fig. S7).

## Discussion

**Figure 6:**
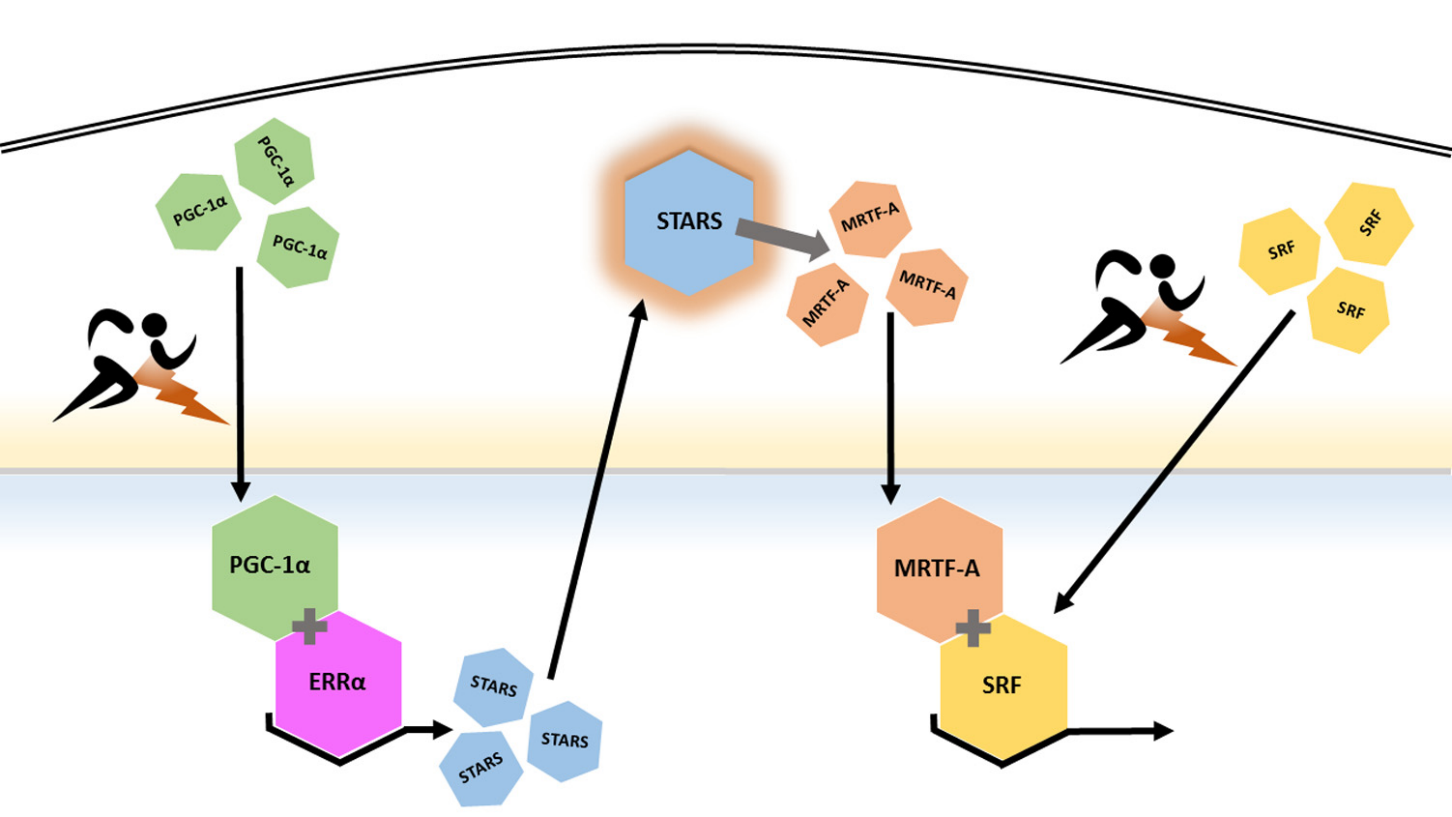
Proposed connection between external stimulus and intracellular response and adaptation via PGC‐1α/ERRα, Stars, and MRTF‐A/SRF. Activated by, among others, mechanical activity, PCG‐1α is transported into the nucleus where, together with ERRα, it coactivates transcription of STARS. STARS protein binds at the I‐band of skeletal muscle freeing MRTF‐A which in turn is transported into the nucleus and together with SRF acts as a cofactor for downstream gene activation (modified after Wallace, 2011).

Taken together, the data presented in this highly controlled intervention study demonstrate that STARS mRNA increased in response to acute endurance exercise (AE). In contrast, no cumulative effect of long‐term endurance training (AE) on STARS expression could be observed. Furthermore, MRTF‐A and SRF, the downstream targets of STARS (see Fig. 6), appear to be temporally activated by acute exercise in a similar manner as STARS. Based on the findings from Arai et al. it is likely that this similar activation pattern is caused by a direct functional connection between them. In the canonic concept of the STARS pathway, STARS, MRTF‐A and SRF protein are directly connected to each other. The STARS protein acts as an agent in the polymerizing reorganization and stabilization of monomeric G‐Actin into F‐Actin (Arai et al. 2002). The depletion of the pool of G‐Actin following this polymerization removes an inhibitory effect on SRF transcriptional activity by enabling the translocation of MRTF‐A into the nucleus (Sotiropoulos et al. 1999). However, it is possible that there is an additional indirect regulation by other factors.

In this study, we show an increased expression of STARS mRNA, no effects on the protein level could be detected, neither following AE cycling, nor LT, consisting of 45 sessions of one‐legged knee extension (connected to a cycle ergometer; see Fig. 1C). Interestingly, both the acute and long‐term training lead to an upregulation of MRTF‐A and most notably SRF mRNA expression, a signaling cascade that would be induced by the presence of STARS protein. Recently, similar results have been reported with increases in SRF mRNA expression without increases in STARS protein expression 2 h following acute resistance exercise in young healthy males (Russell et al. 2017). Lamon et al. (2013) demonstrated a similar effect in long‐term resistance training, but also observed the same effect as response to acute resistance exercise. While based on the mechanics of a cycle ergometer, our one‐legged knee extension machine allows performing a single‐joint isolation exercise recruiting mainly the *M. quadriceps femoris*, which is only a small fraction of total skeletal muscle mass (Janssen et al. 2000). It could be argued that STARS protein expression might require a systemic response, an effect previously demonstrated in PGC‐1*α* (Ydfors et al. 2013). Therefore, one‐legged knee extension exercise alone might not recruit enough muscle mass at an adequate intensity to induce a larger systemic response leading to a change in expression at the protein level. While we demonstrate no significant changes of STARS protein expression within trained or untrained leg, the notion of a possible systemic influence on the expression of STARS protein, but also gene expression is supported by the significant positive intraindividual correlation of changes in STARS protein and mRNA expression between trained and untrained leg in LT (Fig. S6C and D). This suggests a regulation of STARS that might also be dependent on systemic mechanisms. However, considering a protocol recruiting more muscle mass, Lamon et al. (2013) found no changes in protein expression following either an intense lower limb resistance training program or a cycling training program. In addition to this, it has previously been suggested that the translocation of MRTF‐A might also be caused by a system‐wide reduction of monomeric G‐actin and its reorganization into F‐actin (Arai et al. 2002; Kuwahara et al. 2005). This allows for the speculation that the response might be directly correlated with the total amount of muscle activated by a specific training program. Previous mechanistic studies showed that transcriptional activation of SRF does not entirely depend on STARS‐activation, as approximately half of its activation depends directly on RhoA‐mediated signals, which also influence actin polymerization by stabilizing its product, polymerized actin (Arai et al. 2002). This mechanism would explain a regulatory effect of physical activity on MRTF‐A and SRF gene expression in the absence of the STARS protein.

A previous study has demonstrated an increase in SRF protein expression following long‐term (8 weeks) resistance training, consisting of lower body multi‐joint‐exercises (Lamon et al. 2009). That protocol resulted in a significant upregulation of STARS and RhoA mRNA and an induction of MRTF‐A mRNA expression, resulting in increased SRF protein expression. While it remains speculative how RhoA is affected by different exercise and training protocols, it has been demonstrated previously that RhoA alone can facilitate the translocation of MRFT‐A into the nucleus (Miralles et al. 2003). It might therefore be a possibility that RhoA rather than STARS correlates more closely with SRF activity, making it a more important activator of SRF.

Interestingly, it has been shown that three sessions of knee extension resistance training during 1 week lead to an increase in STARS mRNA and protein expression, especially when this training focused on eccentric exercise performance (Vissing et al. 2013). This suggested superior effect of eccentric training on STARS gene expression compared with concentric training might be explained by the damaging nature of eccentric contractions. This structural damaging is known to be caused by the lengthening of muscle fibers under tension and associated with inflammation and damage to muscle proteins and the sarcoplasmic reticulum (Newham et al. 1987; Armstrong 1990; Fridén and Lieber 2001; MacNeil et al. 2010). Taken together, the relatively small muscle mass activated in the knee extension endurance exercise employed in this study, as well as the concentric nature of this exercise, could in part explain the absence of increased protein levels after long‐term training.

The acute effect of endurance exercise on STARS mRNA and protein expression has been examined previously. While mRNA expression seems to commonly be affected (Wallace et al. 2011; Lamon et al. 2013), only one study succeeded to induce STARS protein expression after an acute bout of one‐legged cycling (Wallace et al. 2011). While these studies and the present apply a comparable intensity in percent of $\dot{V}$O_2_peak (60–75%), only Wallace et al. required subjects to cycle until exhaustion whereas other protocols stipulated a specific duration. Previous publications suggest that local, muscular factors rather than central, cardiorespiratory factors are responsible for reaching exhaustion with one‐legged cycling exercise at a given intensity (Andersen and Saltin 1985; Andersen et al. 1985; Rowell et al. 1986). STARS is localized to the I‐band and Z‐disk of the sarcomere (Arai et al. 2002), which also act as important sensors of muscle stretch and contraction (Gautel 2011; Knöll et al. 2011). Therefore, it could be speculated that local muscular exhaustion and the damage to these structures followed by regeneration mechanisms rather than sole mechanical activity or systemic hormonal response might be important for induction of the STARS protein. This is somewhat supported by what seems to be a more profound expression of STARS protein following resistance rather than endurance exercise and training (Lamon et al. 2009, 2013; Wallace et al. 2011; Vissing et al. 2013; Russell et al. 2017). Still, it remains unclear if this is caused by local exhaustion, as explained above, or as a sign of a hypertrophic response of a long‐term resistance training protocol, leading to an increase in muscle cross‐sectional area.

In this study, we report an increase of PGC‐1*α* gene expression and a positive tendency in ERR*α* at the 24‐h time point following 12 weeks of long‐term training. ERR*α* and its transcriptional co‐activator PGC‐1*α* have been shown to be upregulated following exercise (Cartoni et al. 2005; Russell et al. 2005; Perry et al. 2010; Norrbom et al. 2011; Egan et al. 2013; Granata et al. 2016) and also to regulate a whole network of genes involved in skeletal muscle metabolism, mitochondrial function and skeletal muscle mass maintenance (Hock and Kralli 2009). Acute upregulation of PGC‐1*α* mRNA expression has been reported between 4 and 16 h following one exercise bout (Perry et al. 2010; Egan et al. 2013; Gidlund et al. 2015), also an overall tendency for a return to baseline after 24 h (Perry et al. 2010; Gidlund et al. 2015). However, this acute upregulation seemed to significantly decrease after each additional training session in a “stair‐case” type response pattern (Perry et al. 2010). While Perry et al. (2010) demonstrated a complete return to baseline of PGC‐1*α* gene expression at 24 h, it was still somewhat elevated at the same time point in Gidlund et al. (2015), nevertheless with a clear tendency toward baseline.

Taken together, the results of the present and previous studies might suggest that this “stair‐case”‐effect does not necessarily gradually flatten out the acute response of PGC‐1*α* mRNA expression following a long‐term training protocol. Looking at the difference between the exercise protocols of Perry et al. (HIIT cycling) and Gidlund et al. (continuous cycling at 70% $\dot{V}$O_2_peak*)*, exercise intensity might play a role not only in the slightly different time point of return of PGC‐1*α* mRNA expression to baseline, but also in the shape of this “stair‐case”‐effect. While no changes in protein expression could be observed in this study, both Perry et al. and Gidlund et al., report an upregulation of PGC‐1*α* protein expression. The notion that exercise intensity might also play a role in PGC‐1*α* protein expression is further supported by recently published results where acute all‐out cycling sprints, but not moderate‐intensity continuous cycling (63% of W_peak_) were able to induce PGC‐1*α* protein expression (Granata et al. 2016).

Previous results in mice show that this PGC‐1*α* expression is to a large part dependent on systemic *β*‐adrenergic signaling (Miura et al. 2007). Hypoxic stress (Arany et al. 2008) and treatment with the *β*‐adrenergic agonist clenbuterol in mice is also capable of heavily inducing PGC‐1*α* expression (Chinsomboon et al. 2009; Brandt et al. 2017). Furthermore, previously published results demonstrated that PGC‐1*α* protein levels depend on skeletal muscle fiber type (Russell et al. 2003). Taken together, this suggests the notion that PGC‐1*α* expression overall might, to a considerable part, be influenced by systemic factors mediated via the endocrine system or via hypoxia‐induced functions transmitted by the circulatory system. Furthermore, expression could be influenced by basal, fiber‐type specific levels of expression, type of exercise and exercise intensity as well.

However, while there was no significant change within trained or untrained leg, we demonstrate a significant positive intraindividual correlation of changes in PGC‐1*α* protein expression between trained and untrained leg, suggesting a regulation of PGC‐1*α* that is more dependent on a systemic exercise responses. We have previously shown that one‐legged knee extension endurance exercise leads to an increased PGC‐1*α* mRNA expression when blood flow was restricted (Norrbom et al. 2011). This again suggests that some additional exercise intensity is required, especially in a type of exercise that recruits a relatively low amount of muscle mass like one‐legged knee extension to obtain a response in PGC‐1*α* expression. However, while we saw a response in PGC‐1*α* mRNA expression after 45 sessions in the one‐legged long‐term endurance training, additional systemic stress might be required to trigger an increase in protein expression.

To our knowledge, so far all studies examining the STARS pathway used male subjects only. Previously published RNA sequencing data from our long‐term study suggest that roughly 20–25% of the transcripts are differentially expressed between the sexes (Lindholm et al. 2014a). Another study looking at sex differences in skeletal muscle during sprint interval training found that muscle protein synthesis and mitochondrial biogenesis was 35–50% greater in males compared to females (Scalzo et al. 2014). For that reason, in this study, males and females were used as subjects. However, while sex‐specific analyses and correlations were performed in both AE and LT, none of the effects following exercise or training seemed to be linked to sex differences. Nevertheless, while the statistical power indeed was very appropriate in analyses combining the sexes, it should certainly be noted that the validity of conclusions regarding the influence of sex on gene and protein expression in the STARS pathway, whereas statistical power was sufficient, would benefit from an increase in the number of subjects.

Interestingly, in long‐term training, PGC‐1*α* mRNA and protein expression did not correlate with either performance at baseline in the $\dot{V}$O_2_peak‐ or the one‐legged 15‐min‐time‐trial or with absolute improvement in the 15‐min‐time‐trial after the training period. However, it might be argued that the change in performance is explained by the gradual $\dot{V}$O_2_peak‐based adjustment of intensity over the training period. Furthermore, citrate synthase and *β*‐HAD analysis of LT in this study, have been published before showing a significant increase in enzyme activity in the trained leg (Lindholm et al. 2014b). While previously published results suggested a connection between PGC‐1*α* expression and physical performance via mitochondrial mass and oxidative capacity (Lin et al. 2005; Calvo et al. 2008; Norrbom et al. 2010), more recent studies demonstrate that PGC‐1*α* expression in skeletal muscle is not the sole regulator of mitochondrial biogenesis or enhancement of exercise capacity (Rowe et al. 2012; Ballmann et al. 2016). Furthermore, it has been suggested that systemic markers of physical performance like $\dot{V}$O_2_peak in fact do not seem to correlate with biochemical markers locally measured in muscle biopsies (Vollaard et al. 2009). Taken together, our and previously published results suggest that local PGC‐1*α* expression might after all not correlate with systemic markers of physical performance.

While we demonstrate in the present study that STARS, MRTF‐A and SRF mRNA expression largely correlate with one another, it remains unknown whether, or to what degree, this is caused by their direct, canonic relationship through the STARS pathway (Wallace et al. 2011) or if they are simply regulated somewhat independently but simultaneously. While STARS most certainly is involved in actin reorganization (Arai et al. 2002), SRF, and by the same principle MRTF‐A (Sotiropoulos et al. 1999), does not require STARS and might be activated by RhoA itself (Arai et al. 2002), suggesting a more complex regulative network. At protein level in LT, no correlation between PGC‐1*α*, STARS, and SRF expression could be detected, though it has to be noted that none of these factors changed significantly as a result of training. Interestingly, a comparison of changes in protein expression of PGC‐1*α* and STARS in trained and untrained leg in the same individual demonstrated significant correlations in both factors, suggesting a systemic basal level and potentially a “spillover effect”.

To conclude, we show that STARS gene expression can be induced by acute exercise, but long‐term training has no cumulative effect. Furthermore, we showed a somewhat similar regulation pattern of MRTF‐A and SRF acute expression, suggesting a more complex regulative network. Results of protein expression taken together with previously published studies suggest that exercise intensity might play a role in the STARS network. Furthermore, for the first time STARS expression has been analyzed in male and female subjects, revealing no significant influence of sex. Also, while previous studies examined the effect of acute exercise on the STARS pathway in various time points, we demonstrate for the first time a significant influence of exercise on the STARS pathway as early as 30 min after one bout of exercise. Finally, through the usage of intraindividual controls in, we identify what we speculate to be a systemic response of PGC‐1*α* protein expression to long‐term training and a similar systemic mechanism for STARS gene and protein expression.

## Conflicts of Interest

The authors have no conflict of interests to declare.

## Data Accessibility

## Supporting information




**Figure S1.** Individual data of the influence of acute exercise (A and B) and long‐term training (C and D) on STARS mRNA and protein expression.

**Figure S2.** Individual data of upstream regulators of STARS, ERR*α,* and PGC‐1*α* regulation in response to long‐term training, 24 h after the last exercise.

**Figure S3.** Individual data of the regulation of MRTF‐A mRNA expression.

**Figure S4.** Individual data of the influence of acute exercise (A and B) and long‐term training (C and D) on SRF mRNA and protein expression.

**Figure S5.** Correlation analyses of ΔSTARS, ΔMRTF‐A and ΔSRF.

**Figure S6.** Correlation of ΔPGC‐1*α* (A and B) and STARS (C and D) mRNA (A and C) and protein (B and D) expression in trained and untrained leg following long‐term training.

**Figure S7.** Correlation analyses of performance markers and PGC‐1*α* mRNA (A–C) and protein (D–F) expression.
