# Supplementary figures and images for: Expression of striated activator of rho‐signaling in human skeletal muscle following acute exercise and long‐term training
Source: Physiol Rep. 2018 Mar 4;6(5):e13624. doi: 10.14814/phy2.13624 (PMC5835521; doi:10.14814/phy2.13624)

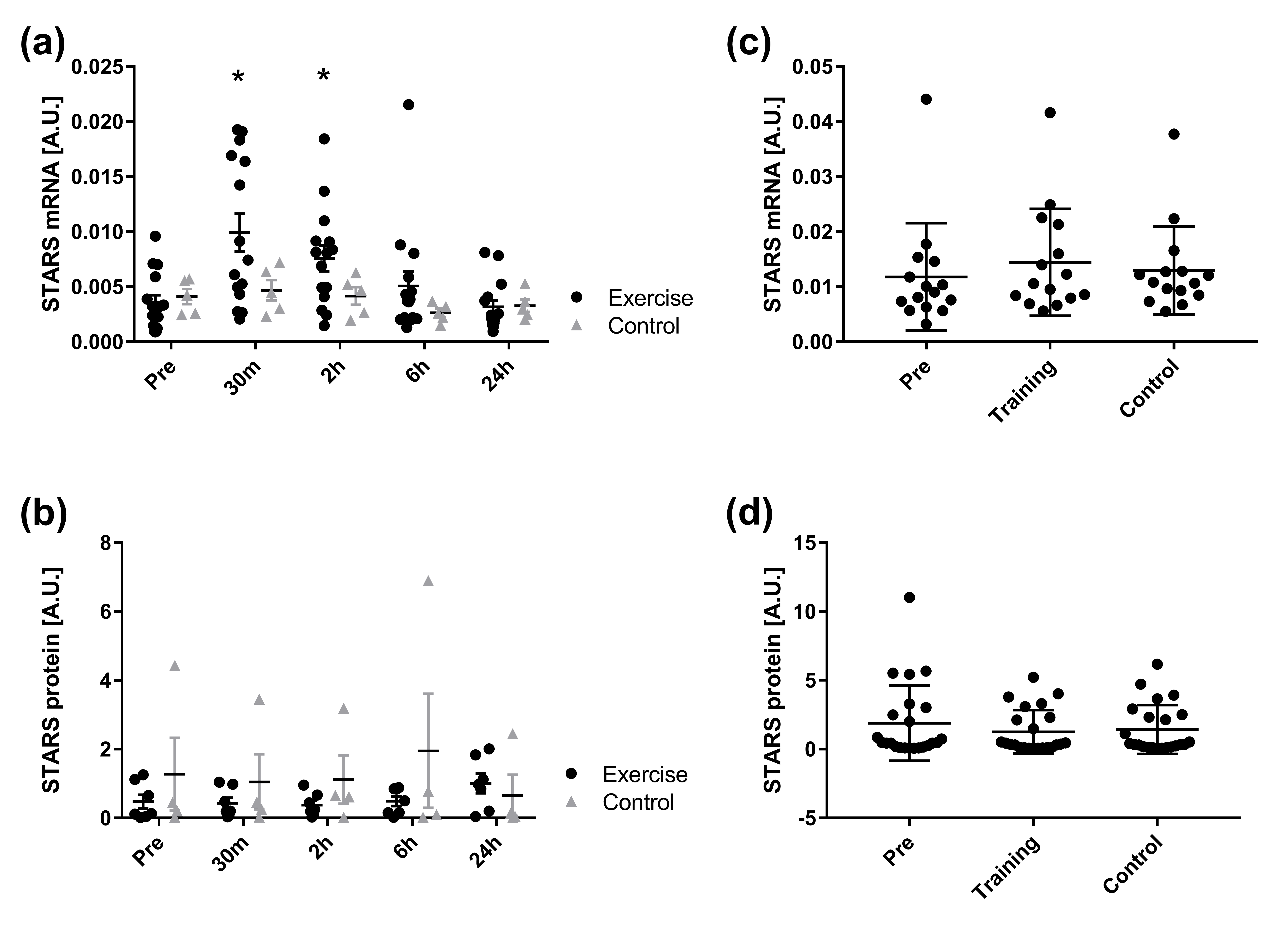

Supplement: Supplementary file 1 — Figure S1. Individual data of the influence of acute exercise (A and B) and long‐term training (C and D) on STARS mRNA and protein expression. [file PHY2-6-e13624-s001.png]

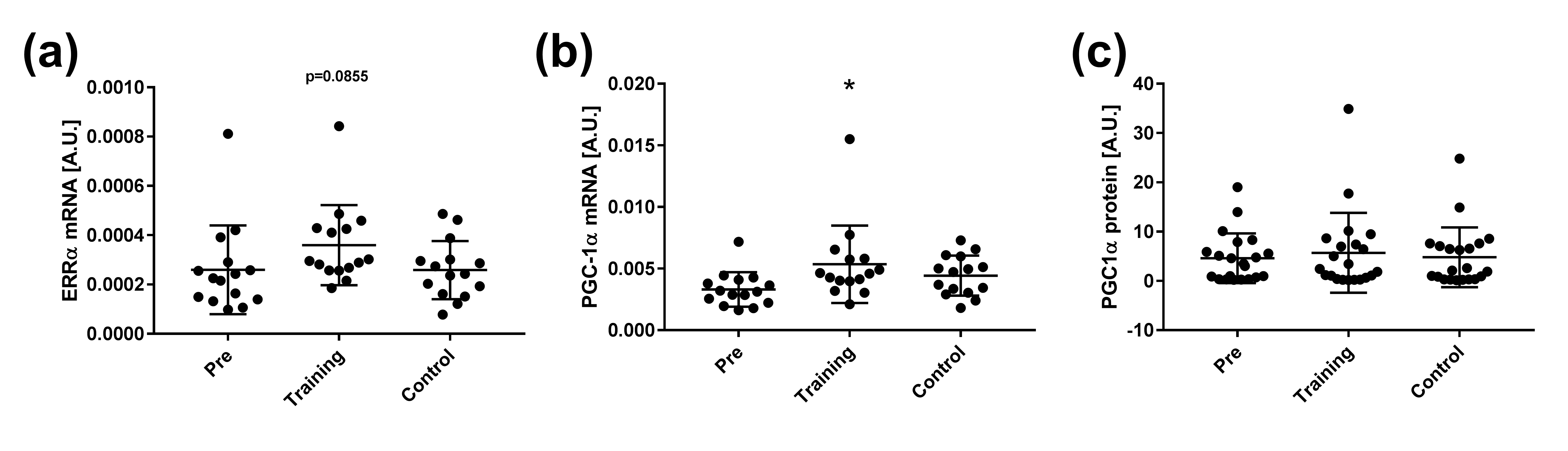

Supplement: Supplementary file 2 — Figure S2. Individual data of upstream regulators of STARS, ERRα, and PGC‐1α regulation in response to long‐term training, 24 h after the last exercise. [file PHY2-6-e13624-s002.png]

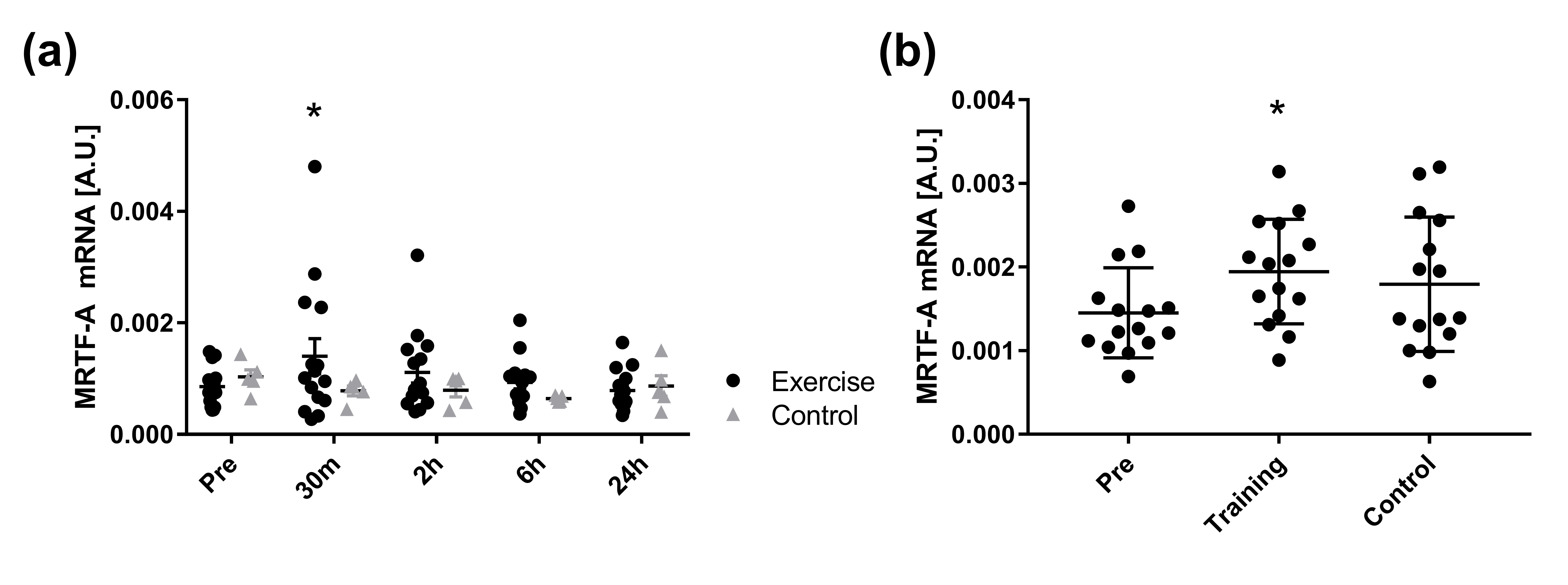

Supplement: Supplementary file 3 — Figure S3. Individual data of the regulation of MRTF‐A mRNA expression. [file PHY2-6-e13624-s003.png]

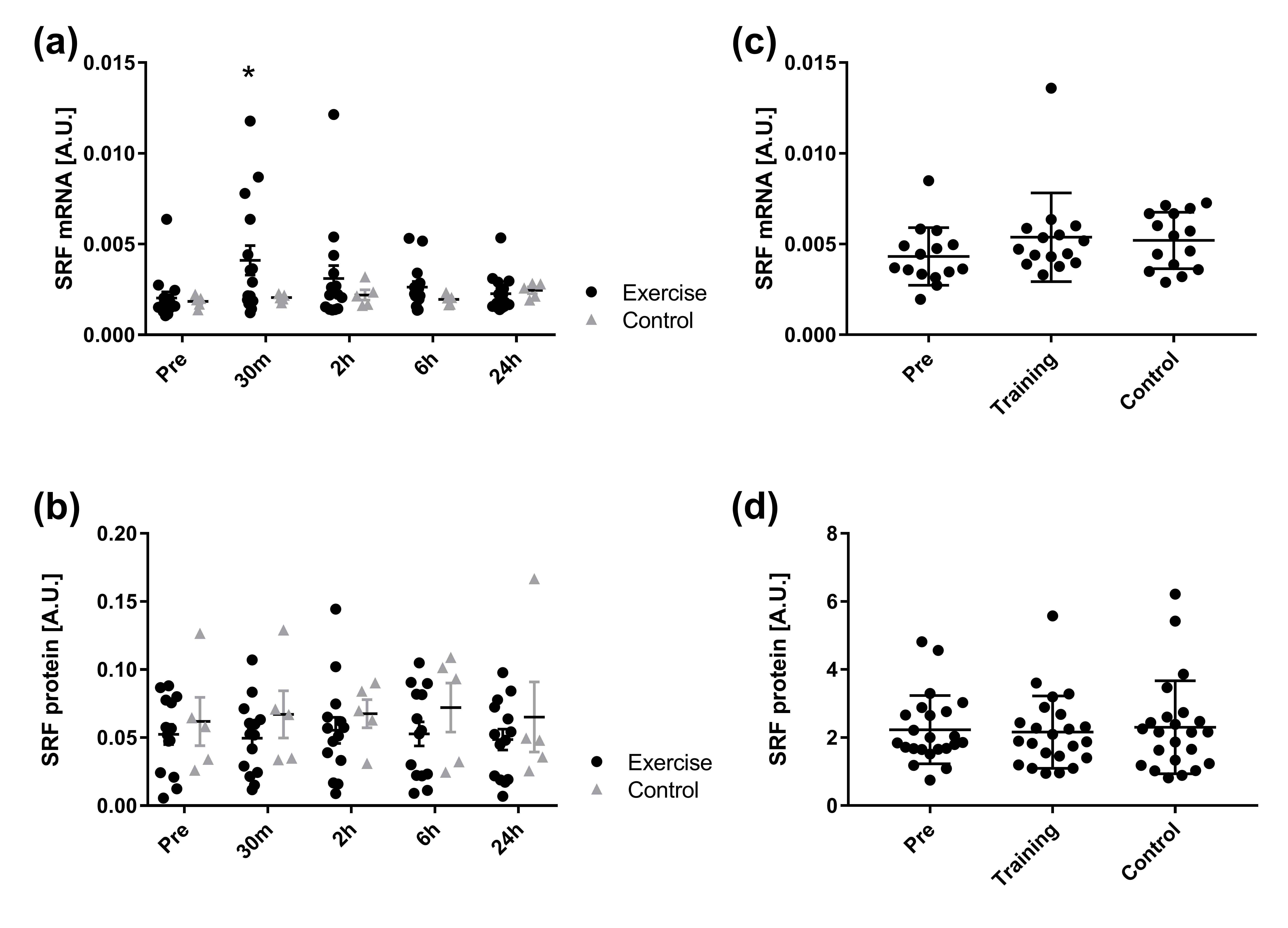

Supplement: Supplementary file 4 — Figure S4. Individual data of the influence of acute exercise (A and B) and long‐term training (C and D) on SRF mRNA and protein expression. [file PHY2-6-e13624-s004.png]

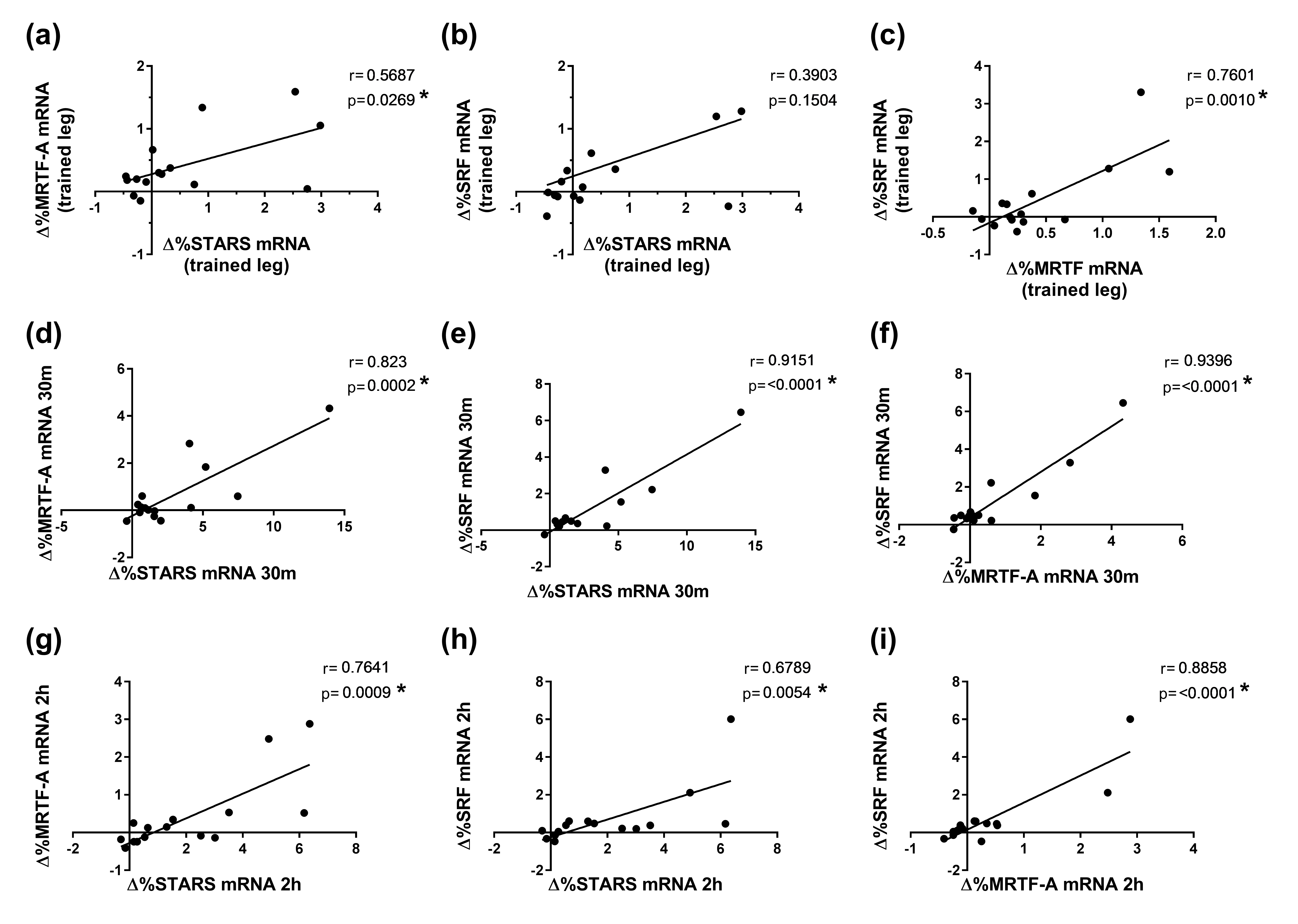

Supplement: Supplementary file 5 — Figure S5. Correlation analyses of ΔSTARS, ΔMRTF‐A and ΔSRF. [file PHY2-6-e13624-s005.png]

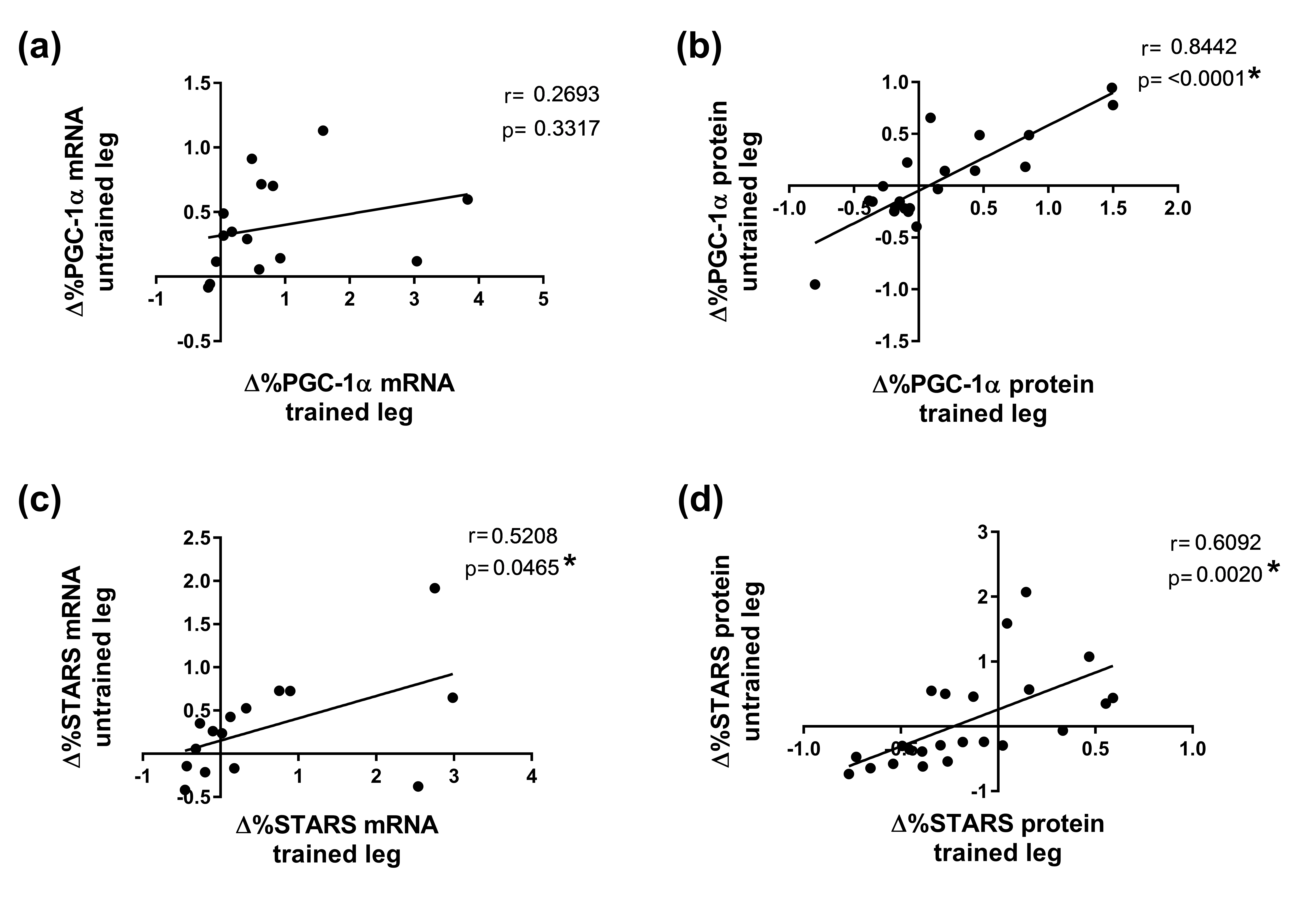

Supplement: Supplementary file 6 — Figure S6. Correlation of ΔPGC‐1α (A and B) and STARS (C and D) mRNA (A and C) and protein (B and D) expression in trained and untrained leg following long‐term training. [file PHY2-6-e13624-s006.png]

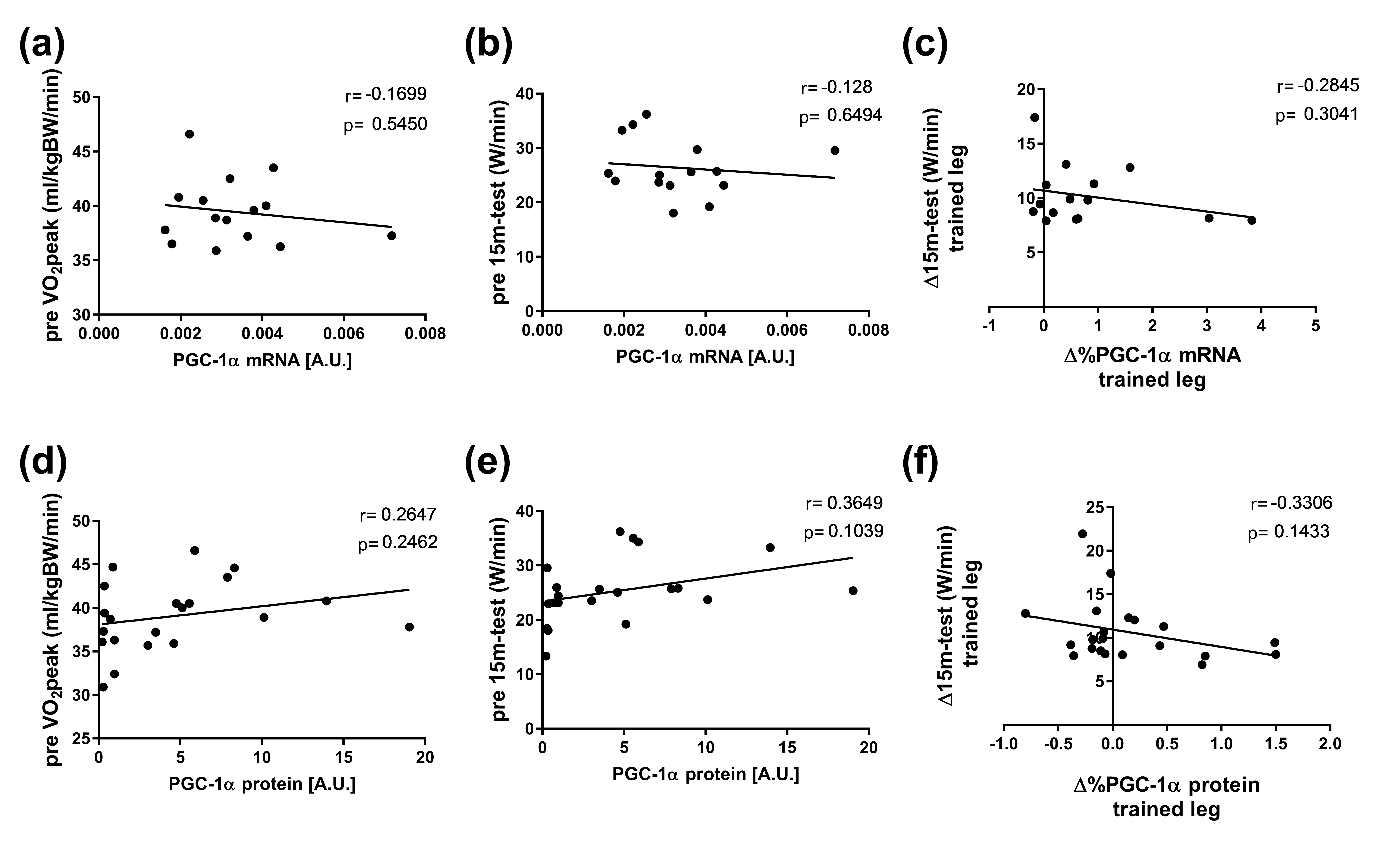

Supplement: Supplementary file 7 — Figure S7. Correlation analyses of performance markers and PGC‐1α mRNA (A–C) and protein (D–F) expression. [file PHY2-6-e13624-s007.png]
