# Supplementary material for: Expression of striated activator of rho‐signaling in human skeletal muscle following acute exercise and long‐term training
Source: Physiol Rep. 2018 Mar 4;6(5):e13624. doi: 10.14814/phy2.13624 (PMC5835521; doi:10.14814/phy2.13624)

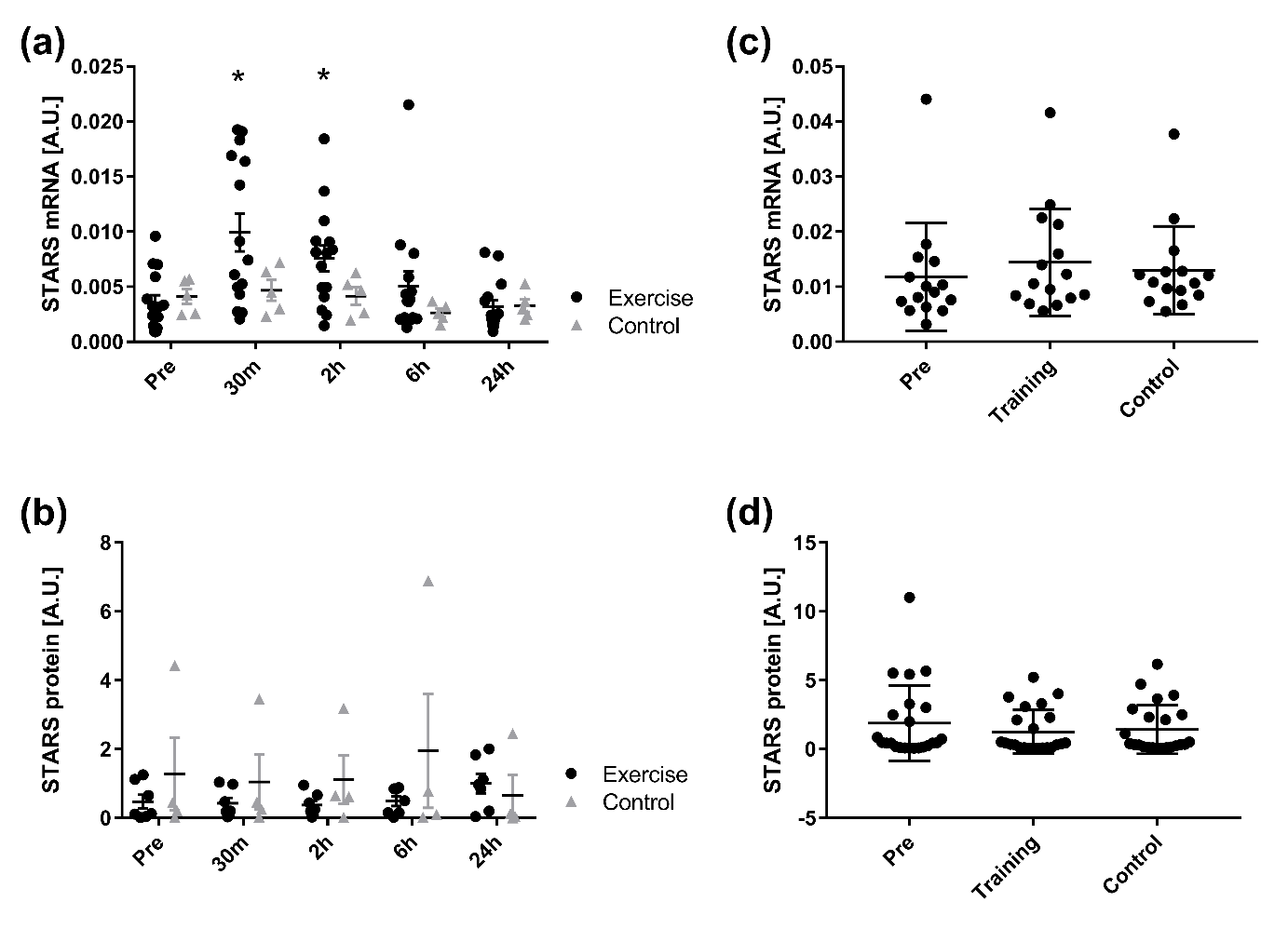


Fig. S1: Individual data of the influence of acute exercise (**a, b**) and long term training (**c, d**) on STARS mRNA and protein expression. **(a):** STARS mRNA is increased significantly as a response to acute exercise at the time points 30 minutes and 2 hours compared to pre time point and returns to baseline after 6 hours. **(b):** STARS protein is increased 24 hours after acute exercise. **(c):** Long term training does not influence STARS mRNA expression 24 hours after the last training session. **(d):** STARS protein expression is not significantly influenced following long term training, 24 hours after the last training session. Data is presented as mean ± SEM. (*=p<0.05)


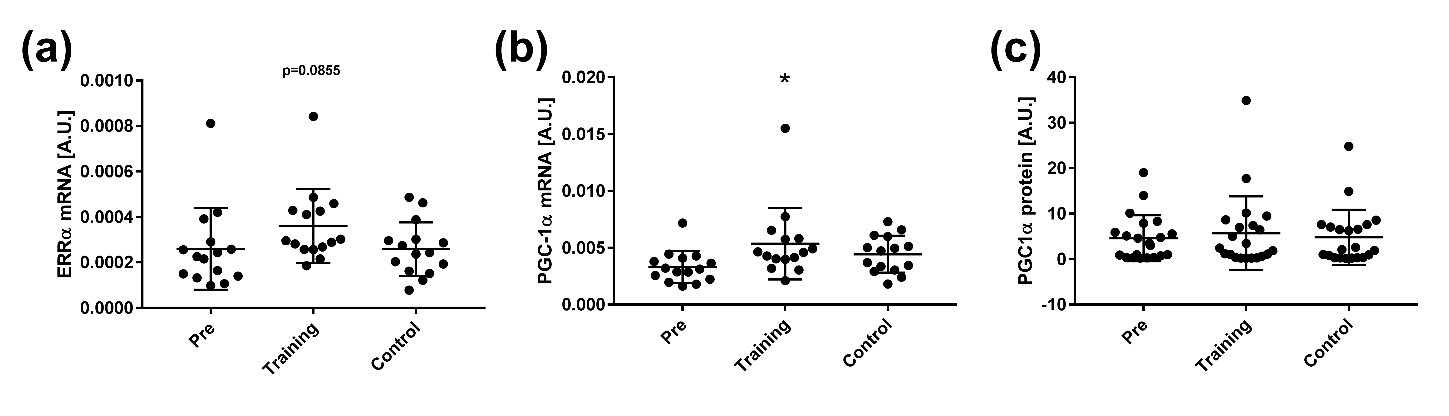


Fig. S2: Individual data of upstream regulators of STARS, ERRα and PGC-1α regulation in response to long term training, 24 hours after the last exercise. **(a):** ERRα is not significantly upregulated in response to training. A slight difference is visible comparing training and control leg after a training period. **(b):** PGC-1α is significantly upregulated in control. A similar but non-significant tendency is also visible in the trainied leg. **(c):** Long term training does not elevate PGC-1α protein expression 24 hours after last exercise. Data is presented as mean ± SEM. (*=p<0.05)


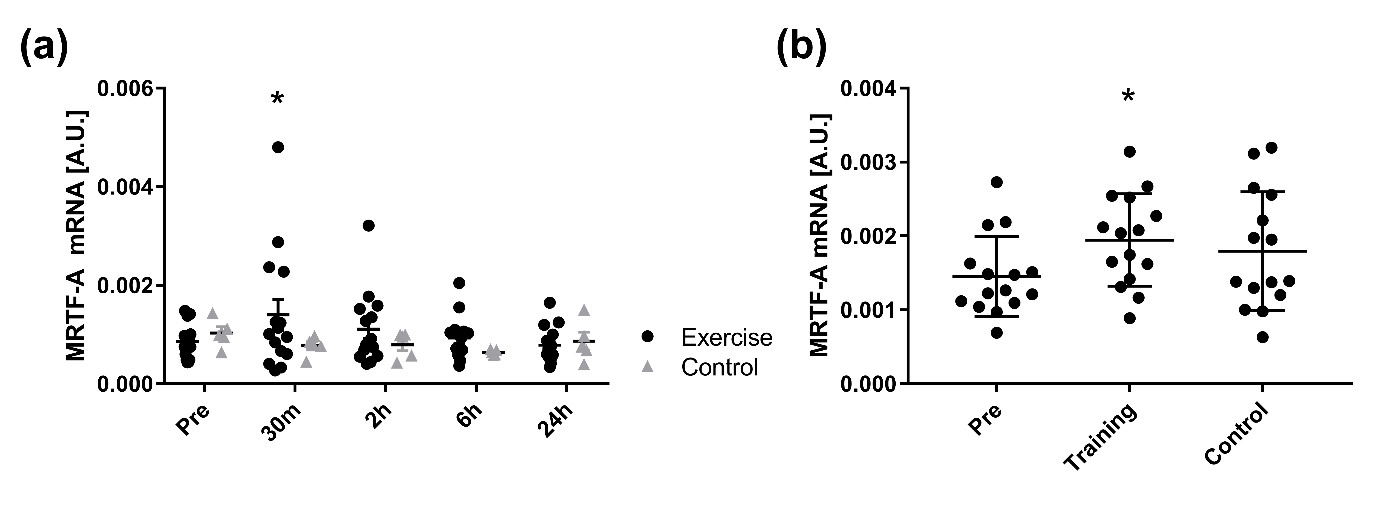


Fig. S3: Individual data of the regulation of MRTF-A mRNA expression. **(a):** MRTF-A mRNA expression is upregulated 30 minutes after end of one acute exercise bout. After that it gradually returns to base line. **(b):** In long term training, 24 hours after last exercise, MRTF-A is significantly upregulated in both, trained leg and control leg. Data is presented as mean ± SEM. (*=p<0.05)


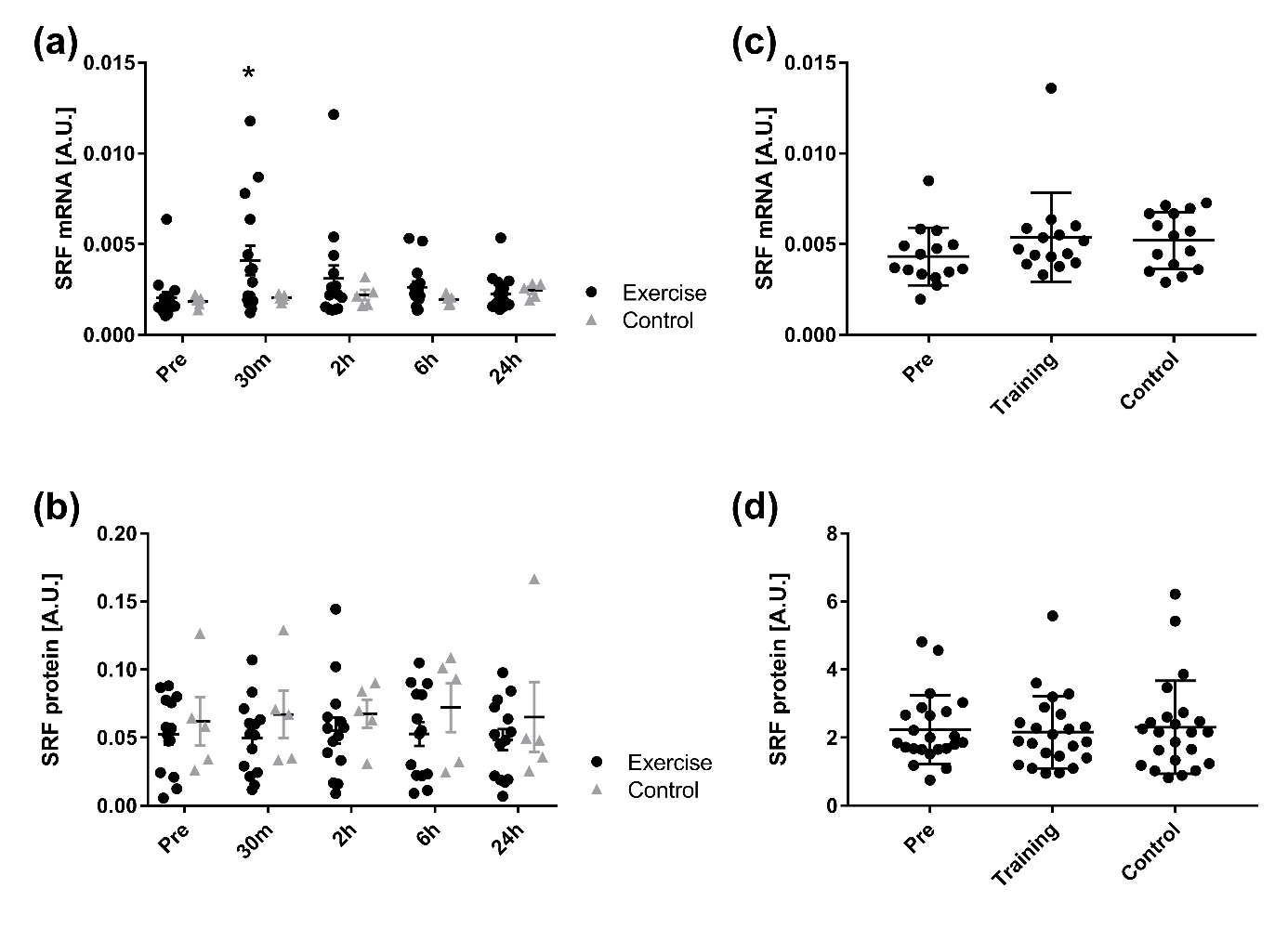


Fig. S4: Individual data of the influence of acute exercise (**a, b**) and long term training (**c, d**) on SRF mRNA and protein expression. **(a):** SRF mRNA is increased significantly as a response to acute exercise at time point 30 minutes compared to pre time point and returns to baseline after that. **(b):** SRF acute exercise protein expression is unchanged. **(c):** Long term training shows the effect of increasing mRNA expression in control. No statistically significant changes are visible in the trained leg. **(d):** SRF protein expression remains unchanged 24 hours following the last exercise in long term training. Data is presented as mean ± SEM. (*=p<0.05)


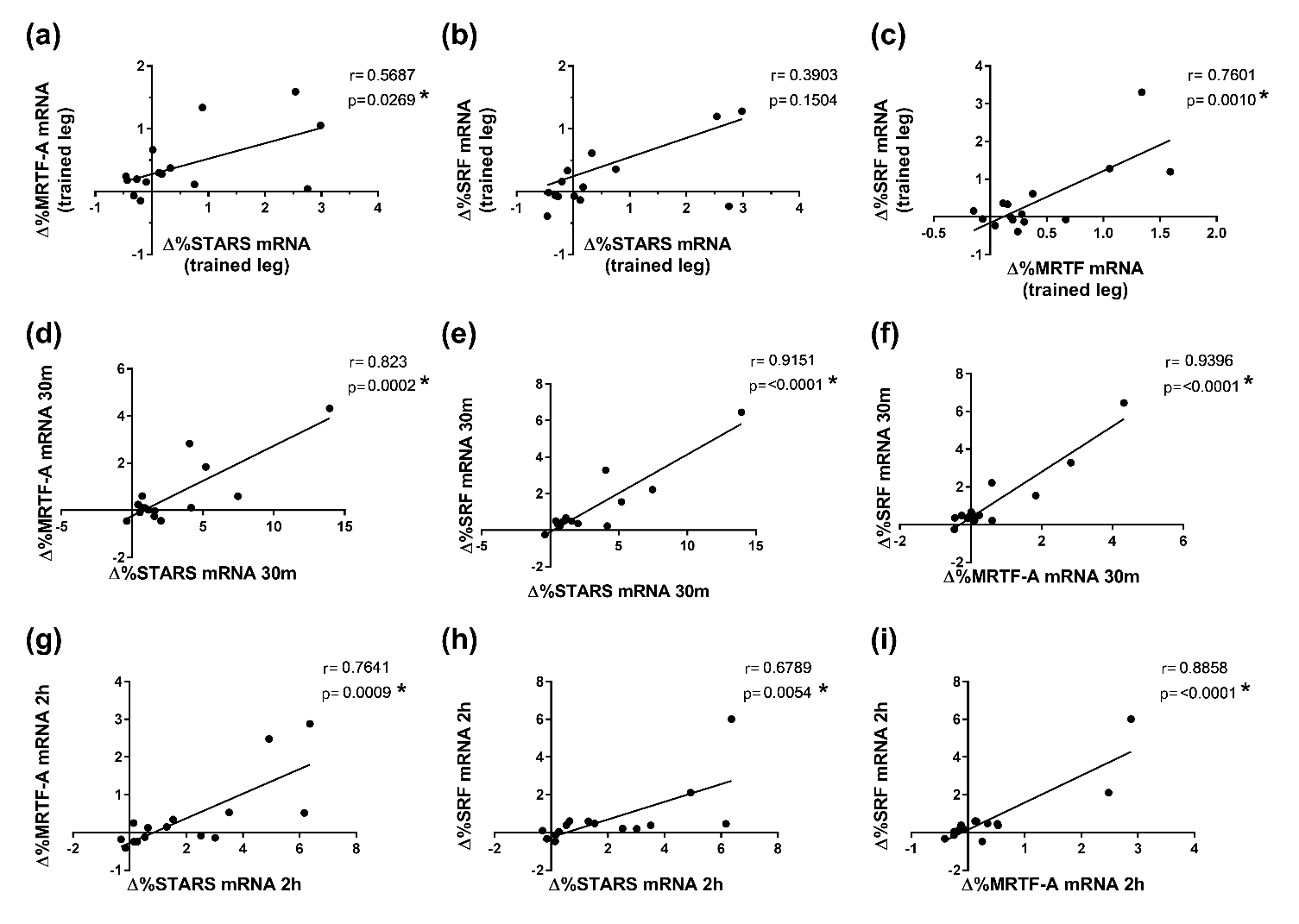


Fig. S5: Correlation analyses of ΔSTARS, ΔMRTF-A and ΔSRF. **(a)-(c):** Correlation of ΔmRNA expression following long-term training. **(d)-(f):** Correlation of of ΔmRNA expression following acute exercise at time-point 30 minutes. **(g)-(i):** Correlation of of ΔmRNA expression following acute exercise at time-point 2 hours. Data is presented as mean ± SEM. (*=p<0.05)


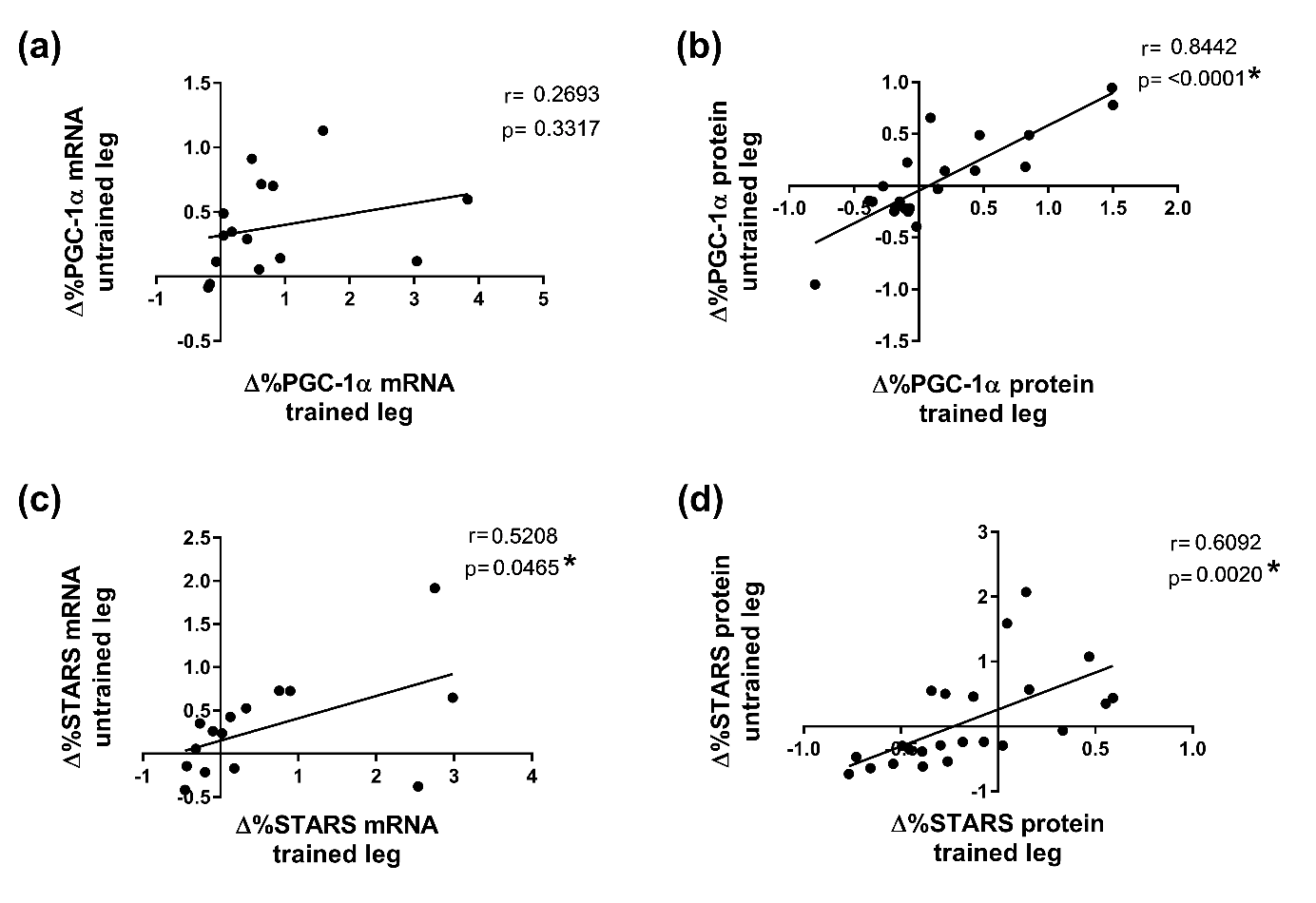


Fig. S6: Correlation of ΔPGC-1α (**a-b**) and STARS (**c-d**) mRNA (**a, c**) and protein (**b, d**) expression in trained and untrained leg following long-term training. Data is presented as mean ± SEM. (*=p<0.05)


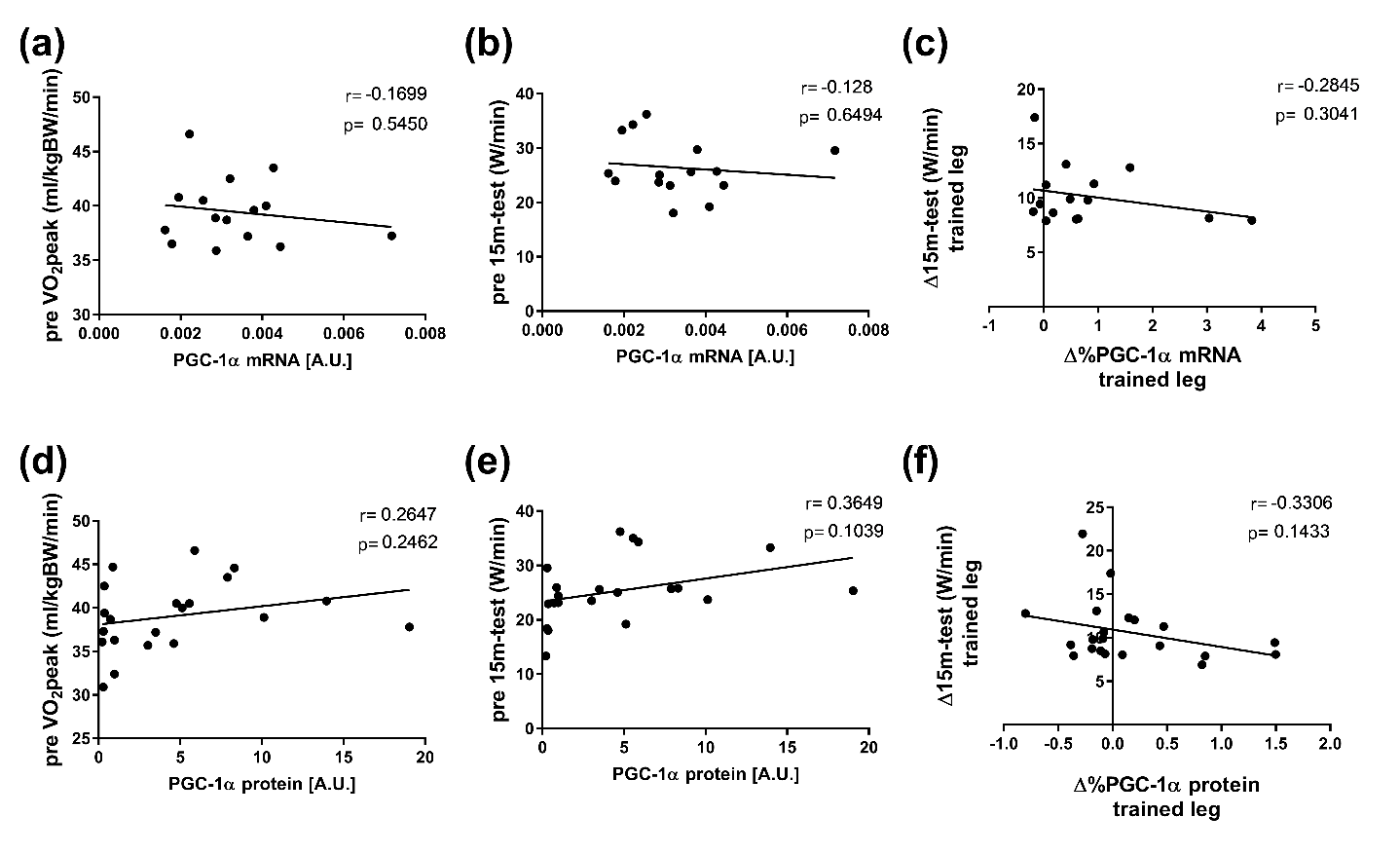


Fig. S7: Correlation analyses of performance markers and PGC-1α mRNA (**a-c**) and protein (**d-f**) expression. **(a),(d):** Absolute PGC-1α expression before training period with V̇O_2_peak results. **(b),(e):** Absolute PGC-1α expression before training period with results in the 15-minutes-time-trial-test. **(c),(f):** Individual ΔPGC-1α expression and Δ15-minutes-test. Data is presented as mean ± SEM.


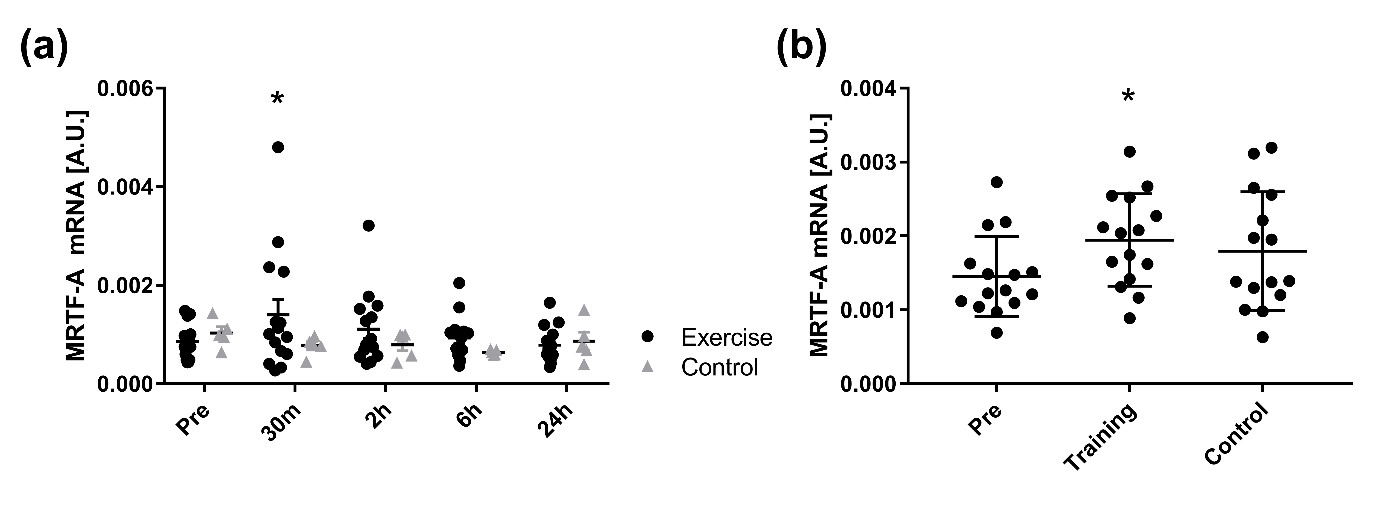

Supplement: Supplementary file 8 [file PHY2-6-e13624-s008.docx]
